# Selection of psychotropic medications for the primary health care essential medicine list: Rationale and process

**DOI:** 10.4102/sajpsychiatry.v24i0.1291

**Published:** 2018-09-20

**Authors:** Lesley J. Robertson, Renee De Waal, Trudy D. Leong

**Affiliations:** 1 Department of Psychiatry, School of Clinical Medicine, University of the Witwatersrand, South Africa; 2 School of Public Health and Family Medicine, Faculty of Health Sciences, University of Cape Town, South Africa; 3 Essential Drugs Programme, National Department of Health, South Africa

## Abstract

**Introduction:**

Equitable access to essential psychotropic medicines at primary level is fundamental to universal health coverage for mental health. It relies upon rational selection, affordable pricing, financial protection and consistent supply systems. Which medicines are selected as essential impacts patient care and the economic sustainability of the health system. Rational selection uses the best available evidence for efficacy, safety and acceptability, and requires the assurance of affordability, obtainability and appropriate usage of the medicines. The process should be reliable, transparent and consultative.

**Aim:**

The aim of this study is to describe the process of rational selection of essential psychotropic medicines at primary health care (PHC) level, using SSRIs as an example.

**Methods:**

Population, intervention, comparison and outcome (PICO) questions were developed for a rapid review of SSRIs for depression and anxiety. PubMed, Trip Database and Cochrane Library were searched for evidence, which was critically appraised (graded as per SORT criteria) and synthesised. Good governance principles were maintained using a consensus decision-making process within the constraints of the PHC Expert Review Committee’s terms of reference, confidentiality and conflict of interest policies. Evidence-based medicine principles were used, considering social values of equity, acceptability, comparative cost and relative budget impact analysis.

**Results:**

The PubMed search for meta-analyses yielded 588 articles, of which 13 met inclusion criteria. An additional 4 meta-analyses were retrieved from the Cochrane Library and 4 from additional reference lists. A second search specifying an HIV population retrieved 43 articles, of which 3 were included. The evidence for efficacy and harm of 24 papers was critically appraised by the committee. Conflicts of interest were declared, and there was appropriate recusal from the final decision-making, which took place in July 2018.

**Conclusion:**

Rapid reviews using an evidence-based medicine framework contributed to the selection of SSRIs for the PHC essential medicine list. This approach is intensive in terms of resources and capacity, requiring adaptation to the South African setting. Important limitations include time pressure and the use of a single reviewer, with possible incomplete database searches and reviewer bias. Nevertheless, the process allows for thorough, consistent and transparent decision-making, and improved capacity is recommended.

